# Light sampling behaviour regulates circadian entrainment in mice

**DOI:** 10.1186/s12915-024-01995-x

**Published:** 2024-09-16

**Authors:** Laura C. E. Steel, Shu K. E. Tam, Laurence A. Brown, Russell G. Foster, Stuart N. Peirson

**Affiliations:** 1 Sir Jules Thorn Sleep and Circadian Neuroscience Institute (SCNi), Kavli Institute for Nanoscience Discovery, Nuffield Department of Clinical Neurosciences, University of Oxford, Oxford, UK; 2 Duke Kunshan University, Kunshan, Jiangsu China; 3 Research IT, University of Oxford, Oxford, UK

**Keywords:** Circadian rhythm, Photoentrainment, Behaviour, Light sampling, Circadian ecology, Nestbox, Cryptochrome

## Abstract

**Background:**

The natural light environment is far more complex than that experienced by animals under laboratory conditions. As a burrowing species, wild mice are able to self-modulate their light exposure, a concept known as light environment sampling behaviour. By contrast, under laboratory conditions mice have little opportunity to exhibit this behaviour. To address this issue, here we introduce a simple nestbox paradigm to allow mice to self-modulate their light environment. Dark nestboxes fitted with passive infrared sensors were used to monitor locomotor activity, circadian entrainment, decision making and light environment sampling behaviour.

**Results:**

Under these conditions, mice significantly reduce their light exposure to an average of just 0.8 h across a 24 h period. In addition, mice show a distinct pattern of light environment sampling behaviour, with peaks at dawn and dusk under a ramped light dark cycle. Furthermore, we show that the timing of light environment sampling behaviour depends upon endogenous circadian rhythms and is abolished in mice lacking a circadian clock, indicating a feedback loop between light, the circadian clock and behaviour.

**Conclusions:**

Our results highlight the important role of behaviour in modifying the light signals available for circadian entrainment under natural conditions.

**Supplementary Information:**

The online version contains supplementary material available at 10.1186/s12915-024-01995-x.

## Background

Due to the rotation of the earth on its axis, almost all life has evolved under rhythmic cycles of light and dark. As a result, most organisms have evolved circadian rhythms—endogenous ~ 24 h rhythms in physiology and behaviour that persist even under constant conditions [1, 2]. When aligned to the external environment these internal biological clocks provide a selective advantage by enabling organisms to anticipate predictable daily changes and align internal physiology and behaviour to the varied demands of the solar day [3–7]. Circadian rhythms rarely have a period of precisely 24 h, and in mice they are on average 23.7 h [8, 9]. Adjustment of the circadian clock by external time cues (termed zeitgebers) is therefore necessary to maintain appropriate alignment between the internal clock and the external environment [10]. Whilst there is evidence that food availability [11] and temperature [12] are important zeitgebers in some species, in mammals light is the primary zeitgeber—a process termed photoentrainment.

Light is detected by retinal photoreceptors [13, 14], including the visual rods (λmax = 498 nm) and cones (S-cones, λmax = 360 nm; M-cones, λmax = 508 nm) as well as the non-visual photopigment melanopsin (λmax = 480 nm) [14]. Information from the retina is sent to the suprachiasmatic nuclei (SCN) in the anterior hypothalamus [15, 16] where cell-autonomous rhythms are generated by an molecular transcriptional-translational feedback loop [17–19]. Circadian clocks are also found in cells throughout the body and the SCN acts as a pacemaker to coordinate these peripheral clocks [20], although many can also entrain independently [21–23].

Much of our knowledge of photoentrainment comes from the study of rodent models under laboratory conditions [24]. However, the laboratory light environment is highly simplified compared to the natural light environment in which the laboratory mouse (*Mus musculus*) and other mouse species live in the wild, and under which their circadian system evolved [25, 26]. In nature, both the intensity and spectrum of light change predictably across the 24 h period as a function of solar angle. Intensity can range from 0.001 photopic lux on a starlit night to over 100,000 photopic lux on a sunny day, and light at twilight (dawn and dusk) is short-wavelength enriched due to both atmospheric absorption and scatter [27–29]. However, in the laboratory, mice are generally housed under 12 h:12 h light/dark (LD) cycles of broad spectrum white light sources, which may be a simple on/off square wave or involve ramped light transitions [14].

The way in which an animal interacts with the light environment is also more complex under natural conditions. In the wild, mice build underground burrows [30]—a behaviour that has also been demonstrated under semi-natural [31] and laboratory environments [32, 33], and which allow animals to self-modulate their light exposure across the day. The motivation to move in and out of a burrow, or to the burrow entrance, is unlikely to solely be light. Rather, a balance of temperature considerations, food availability, predation risk and activity of conspecifics may drive this behaviour [34, 35]. However, light exposure is an important consequence, and this has been termed light sampling behaviour [36–38]. In this way, behaviour is an important factor in determining the timing, intensity and spectrum of light available for photoentrainment [39]. Under laboratory conditions this is often overlooked, with mice typically having little opportunity to self-modulate their light exposure [8].

It has been clearly documented that circadian behaviour is very flexible [40]. For example, mice show reliable nocturnal activity under laboratory conditions. However, when environmental conditions vary in a more naturalistic way, diurnal activity has been observed—such as under low night time temperatures [41, 42] and conditions of limited food availability [43, 44]. This does not result from a change in the phase of the underlying SCN clock, but may reflect independent entrainment of peripheral clocks which facilitate flexibility [23]. Over-standardisation of environmental conditions has been suggested to result in local truths with little external validity [23, 45–49]. As a result, a more naturalistic approach to research – studying physiology and behaviour under conditions in which species evolved—may benefit many fields, from neuroscience [50] to molecular biology [51].

Here we provide laboratory mice (*Mus musculus)* with dark nestboxes and therefore the opportunity to self-modulate their light exposure, allowing circadian entrainment to be studied under more naturalistic conditions. It is possible that mice bred in the laboratory display behavioural differences to those living in the wild [52], but they serve as a model. These behavioural aspects of photoentrainment have only been explored in a few studies previously—in mice [53], hamsters [54] and flying squirrels [36, 37]. Whilst it has been suggested that self-modulating light exposure does not have a significant effect on photoentrainment in mice [53], this has not been explored under more naturalistic conditions with gradual twilight transitions. Here we show that when given the choice, mice will significantly reduce their daily light exposure and exhibit a distinct pattern of light environment sampling behaviour, with peaks at twilight. Furthermore, the timing of this light environment sampling behaviour depends upon endogenous circadian rhythms and is abolished in mice lacking a circadian clock (*Cry1*^*−/−*^*,Cry2*^*−/−*^). These data illustrate the importance of light environment sampling behaviour in modulating photoentrainment.

## Results

### Nestbox availability significantly reduces daily light exposure

**Fig. 1:**
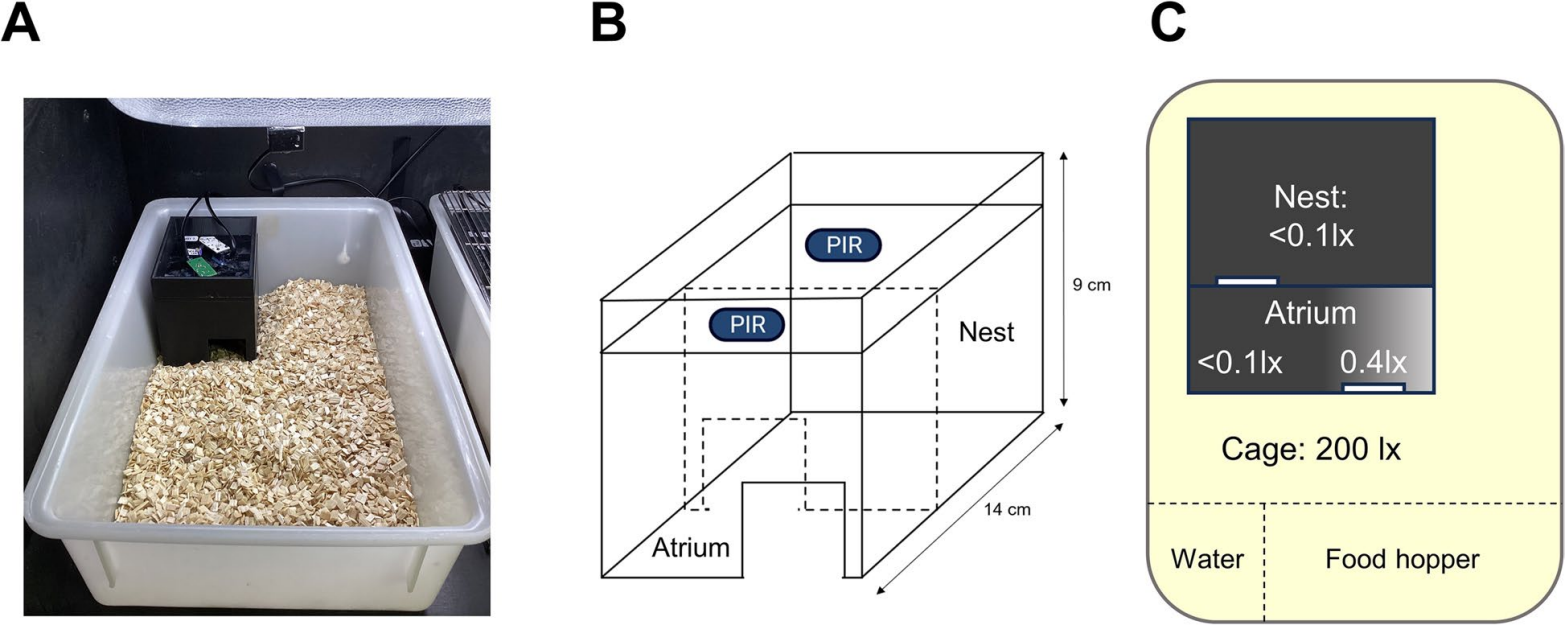
Experimental setup. **A** Photo of nestbox in-situ, with passive-infrared sensor (PIR) above cage. **B** Schematic of nestbox design (not to scale), with PIRs. **C** Light levels across nestbox and cage, measured using an XL-500 BLE Spectroradiometer (NanoLambda, Korea), with location of water bottle and food hopper marked

**Fig. 2:**
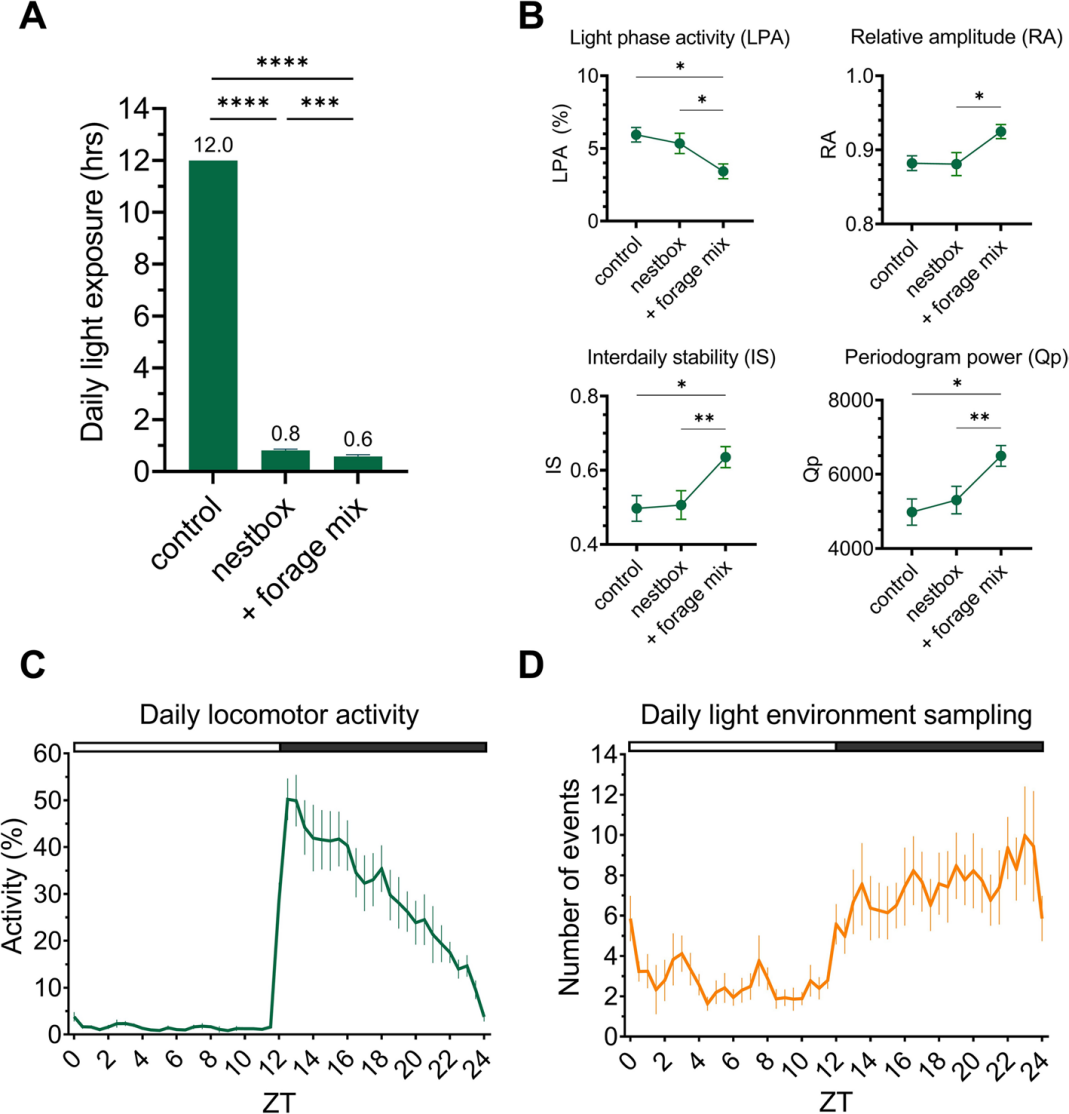
Effects of nestbox availability on light exposure and behaviour. **A** Daily light exposure (hrs) of animals across experimental conditions. **B** Key circadian entrainment parameters across experimental conditions. The combined nestbox and forage mix condition is referred to as ‘ + forage mix’ (A, B). **C** Daily locomotor activity profile of animals housed with a nestbox, calculated using the main cage PIR. **D** Daily light environment sampling profile. White and black bar shows timing of light and dark, respectively (C, D). All results reported as mean across mice and days, ± SEM. **** *p* < 0.001, ** *p* < 0.01, * *p* < 0.05, between condition comparisons

10 out of 12 C57BL/6J animals routinely used the nestbox (Additional file 1: Fig. S1), thereby reducing their daily light exposure. Daily light exposure was significantly different between the control and nestbox condition [t(9) = 235.2, *p* < 0.0001], and between the control and nestbox + forage mix (‘ + forage mix’) condition [t(9) = 195.6, *p* < 0.0001; one-sample t-test against a control mean of 12.0 h], with mice reducing their average daily light exposure to 0.8 h under the nestbox condition, and to 0.6 h under the nestbox and forage mix condition (Fig. 2A). The addition of forage mix in addition to a nestbox also significantly reduced light exposure, compared to the nestbox only condition [t(9) = 4.8, *p* < 0.0009; paired t-test]. This difference is likely to result from mice being able to take food back to the nestbox under the forage mix condition, rather than having to leave the nestbox to feed from the external food hopper (Fig. 1C) under the nestbox only condition.

### Nestbox availability with forage mix significantly affects measures of circadian disruption

Based upon the main cage sensor, the addition of a nestbox in combination with forage mix resulted in significant differences in metrics of circadian rhythm disruption in C57BL/6J mice, compared to under the control or nestbox only conditions (Fig. 2B). Experimental condition (control, nestbox, nestbox + forage mix (‘ + forage mix’)) had a significant effect on light phase activity [F(2.2, 20.1) = 11.3, *p* = 0.0004], relative amplitude [F(2.4, 21.6) = 6.8, *p* = 0.0034], inter-daily stability [F(2.2, 19.4) = 10.8, *p* = 0.0006] and periodogram power [F(1.9,16.7) = 11.4, *p* = 0.0009], as tested by a one-way repeated-measures ANOVA. Further circadian metrics were calculated and are reported in the supplementary material (Additional file 2: Fig. S2). Across all parameters, post-hoc Tukey tests demonstrated significantly more robust activity patterns under the nestbox + forage mix condition compared to the control or nestbox only conditions, as evidenced by decreased light phase activity (control vs nestbox + forage mix, *p* = 0.0411; nestbox vs nestbox + forage mix, *p* = 0.01), increased relative amplitude (nestbox vs nestbox + forage mix, *p* = 0.0198), inter-daily stability (control vs nestbox + forage mix, *p* = 0.0335; nestbox vs nestbox + forage mix, *p* = 0.0089) and periodogram power (control vs nestbox + forage mix, *p* = 0.0241; nestbox vs nestbox + forage mix, *p* = 0.0055) [55]. These findings demonstrate how adding environmental enrichment can modify commonly calculated metrics of circadian behaviour.

### Daily locomotor activity does not reflect daily light environment sampling behaviour

The daily locomotor activity profile of C57BL/6J mice (Fig. 2C) illustrates low activity levels across the light phase, followed by a peak in activity at the onset of the dark phase, with levels declining across the rest of the night. Daily light environment sampling events (Fig. 2D) (defined as movement from the nest to the atrium) also show a rhythmic pattern, with lower levels during the light phase and higher levels at night. However, it differs to locomotor activity with light environment sampling events remaining constant across the dark phase. This pattern is consistent across days, under all conditions (Additional file 3: Fig. S3). Differences in the pattern of locomotor activity and light environment sampling behaviour are further demonstrated when sex differences are considered (Additional file 4: Fig. S4). Females show significantly higher locomotor activity, under both a 12:12 h (Additional file 4: Fig. S4, A) [F(1,10) = 14.1, *p* = 0.0037] and 12:2:8:2 h LD cycle (Additional file 4: Fig. S4, C) [F(1,10) = 6.1, *p* = 0.0332]. However, there is no significant main effect of sex on light environment sampling behaviour, under either a 12:12 h (Additional file 4: Fig. S4, B) [F(1,8) = 1.4, *p* = 0.2639] or 12:2:8:2 h LD cycle (Additional file 4: Fig. S4, D) [F(1,8) = 0.9, *p* = 0.3647] (two-way repeated-measures ANOVA and post-hoc Bonferroni test).

### Under a ramped LD cycle mice show peaks in light environment sampling behaviour at twilight

**Fig. 3:**
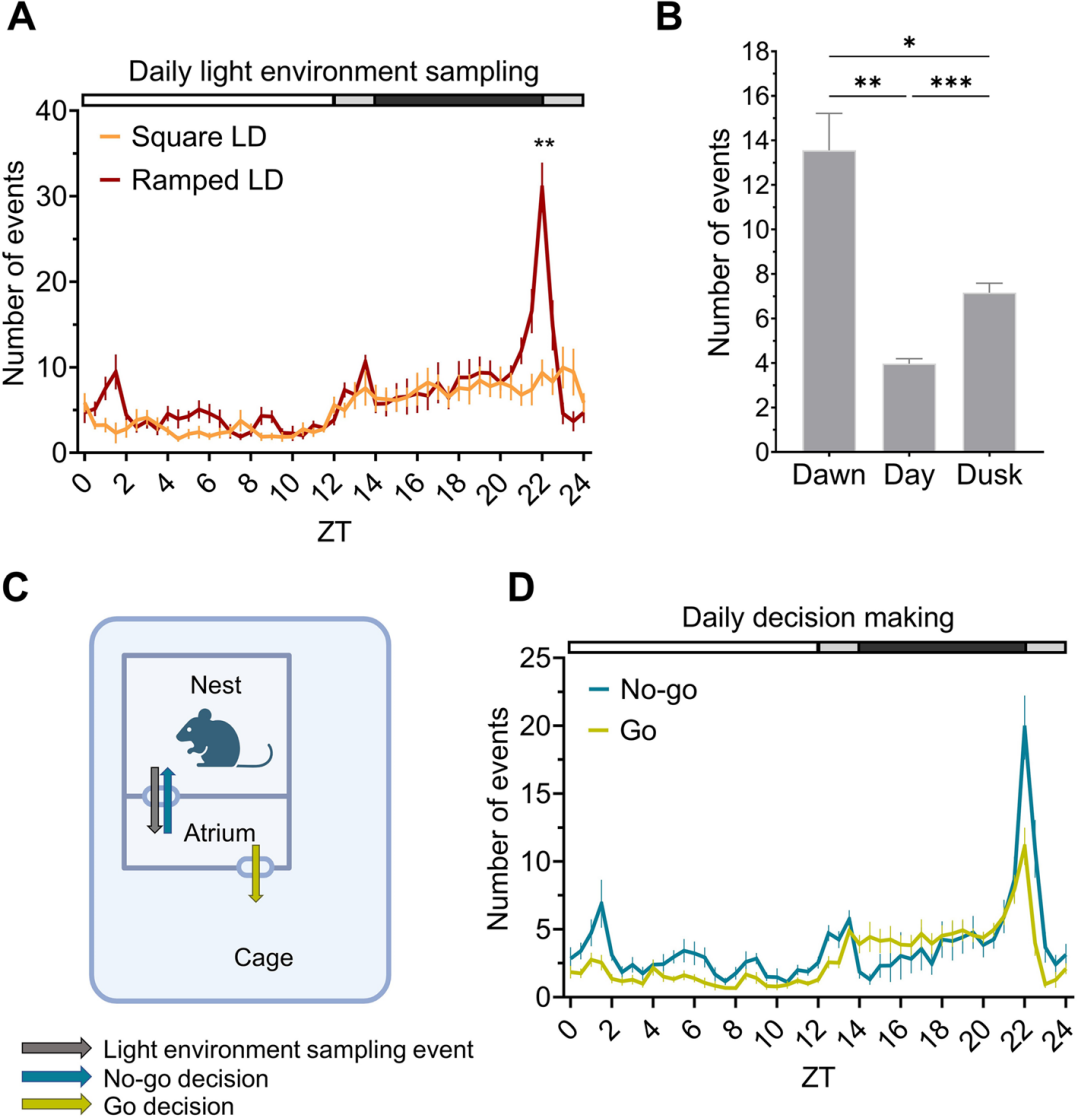
Effects of a ramped LD cycle on light sampling behaviour. **A** Daily light environment sampling profile under a square 12:12 h LD cycle (green) and a ramped 12:2:8:2 h LD cycle (blue). **B** Number of light environment sampling events occurring during dawn (ZT22-0), the day (ZT0-12) and dusk (ZT12-14), under a ramped 12:2:8:2 h LD cycle. **C** Schematic demonstrating “no-go” or “go” decision making following light environment sampling behaviour. **D** Daily decision making profile showing “no-go” (red) and “go” (green) decisions under ramped 12:2:8:2 h LD cycle. White, grey and black bar shows timing of light, light ramp and dark, respectively (A, D). All results reported as mean across mice and days, ± SEM. **** *p* < 0.001, ** *p* < 0.01, * *p* < 0.05, between condition comparisons

Changing the LD cycle to a ramped 12:2:8:2 h LD cycle results in comparable locomotor activity patterns in C57BL/6J animals as under a square wave 12:12 h LD cycle, in both sexes (Additional file 4: Fig. S4A, C). However, a ramped LD cycle significantly alters the pattern of light environment sampling behaviour across time (Fig. 3A), with a significant main effect of light condition [F(1,9) = 7.3, *p* = 0.0244] and ZT [F(3.5,31.7) = 14.0, *p* < 0.0001] being observed, as well as a significant light condition x ZT interaction [F(4.7,42.4) = 6.7, *p* = 0.0002, two-way repeated measures ANOVA (Fig. 3A)]. A clear peak in light environment sampling events was seen at ZT22 under the ramped LD cycle compared to the square LD cycle (*p* = 0.0023, post-hoc Bonferroni test), immediately following the start of the light-on ramp (corresponding to ‘dawn’). This pattern is consistent across sexes (Additional file 4: Fig. S4D). To explore this difference further, the mean number of light environment sampling events occurring during dawn (ZT22-0), the day (ZT0-12) and dusk (ZT12-14) were calculated (Fig. 3B). Night time (ZT14-22) was not included since there is no light at this time to sample. A significant effect of time of day on number of events was observed [F(2.0,17.8) = 16.4, *p* < 0.0001, one-way repeated-measures ANOVA]. A post-hoc Tukey test demonstrated significantly higher levels of crepuscular activity shown by more light environment sampling events at twilight compared to the day [dawn vs. day (*p* = 0.0014), dusk vs. day (*p* = 0.0005)]. Interestingly, more light environment sampling was seen at dawn than dusk (*p* = 0.0157).

### The nestbox paradigm allows for quantification of decision making behaviour, under a long-term home-cage experiment

Following a light environment sampling event (movement from the nest to the atrium) an animal can either move into the cage (classified as a “go” decision) or return to the nest (classified as a “no-go” decision) (Fig. 3C). Categorising decision making following light environment sampling events under a ramped LD cycle (Fig. 3D) demonstrates that the peak in light environment sampling events at dawn can largely be explained by an increase in “no-go” decisions, with approximately twice as many “no-go” transitions as “go” decisions. Whilst no significant main effect of decision route was observed [F(1,9) = 3.7, *p* = 0.0081], there was a significant main effect of ZT [F(4.4,39.8) = 14.0, *p* < 0.0001] and a significant decision route x ZT interaction [F(3.7,33.5) = 7.5, *p* = 0.0003, two-way ANOVA and post-hoc Bonferroni test]; the latter demonstrating that, as expected in a generally nocturnal, photophobic species, the ratio of “no-go” to “go” decision routes changes across time, with “no-go” decisions being higher during the light phase (ZT22-14), and “go” decisions increasing at dark onset (ZT14-18).

### Using a nestbox may not significantly contribute to thermoregulation

Thermal imaging (FLIR one pro, Teledyne FLIR) of cages with a nestbox recently removed show a 0.3 °C higher temperature in the location of the nestbox, compared to the main cage, when no animal has been nesting in the cage (Additional file 5: Fig. S5A), and a 3.5 °C increase when an animal has been recently using the nestbox (Additional file 5: Fig. S5B). This is comparable to the 4.2 °C increase in temperature in a nest built without a nestbox compared to the main cage (Additional file 5: Fig. S5C), suggesting that building a nest within a nestbox does not dramatically influence thermoregulation.

### Mice lacking a circadian clock use the nestbox less than wildtype controls

**Fig. 4:**
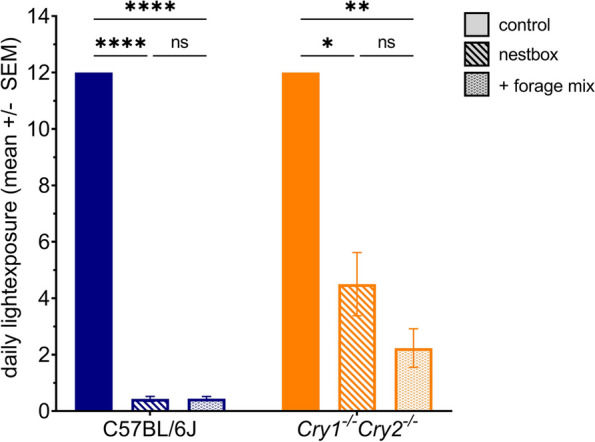
Effects of nestbox availability on daily light exposure in C57BL/6J and *Cry1*^*−/−*^*Cry2*^*−/−*^ animals. Daily light exposure (hrs) perceived by C57BL/6J (blue) and *Cry1*^*−/−*^*Cry2*^*−/−*^ animals (orange) under different conditions. The combined nestbox and forage mix condition is referred to as ‘ + forage mix’. All results reported as mean across mice and days, ± SEM. **** *p* < 0.001, ** *p* < 0.01, * *p* < 0.05, between condition comparisons

To examine the role of circadian rhythms in determining the timing of light environment sampling behaviour, mice lacking a circadian clock (*Cry1*^*−/−*^*,Cry2*^*−/−*^*)* and congenic C57BL/6J controls were tested under the same paradigm as the first C57BL/6J light sampling experiment*.* Fewer *Cry1*^*−/−*^*,Cry2*^*−/−*^ mice (3 out of 6 animals) routinely used the nestbox compared to C57BL/6J WT counterparts (all 6 animals) (Additional file 6: Fig. S6). This difference may relate to attenuated novelty-induced locomotor activity levels in *Cry1*^*−/−*^*,Cry2*^*−/−*^ [56] and altered photic sensitivity [57–60]; highlighting the sensitivity of our paradigm to demonstrate differences in behavioural and physiological phenotypes. Of the mice that did use the nestbox, both *Cry1*^*−/−*^*,Cry2*^*−/−*^ mice and C57BL/6J WT counterparts (Fig. 4) reduced their daily light exposure when a nestbox was available [significant main effect of condition, F(1.2,8.5) = 8, *p* < 0.0001, two-way repeated-measures ANOVA with Tukey–Kramer post-hoc test (C57BL/6J control vs. nestbox, *p* < 0.0001; *Cry1*^*−/−*^*,Cry2*^*−/−*^ control vs. nestbox, *p* = 0.0391)]. However, *Cry1*^*−/−*^*,Cry2*^*−/−*^ mice reduced their daily light exposure less than the C57BL/6J WT counterparts (Fig. 4), to an average of 4.5 h compared to 0.4 h under the nestbox condition, respectively. This trend was reflected in a significant main effect of genotype on daily light exposure [F(1,7) = 24.9, *p* = 0.0016]. Forage mix in addition to a nestbox did not further significantly reduce daily light exposure in C57BL/6J mice, as in the previous C57BL/6J only study, remaining at 0.4 h in both conditions (Fig. 4). This may result from a floor effect, since 0.4 h is already lower than the 0.6 h of daily light exposure exhibited by mice under the nestbox and forage mix condition in the C57BL/6J only study (Fig. 2A). However, in *Cry1*^*−/−*^*,Cry2*^*−/−*^ mice, forage mix availability reduced daily light exposure, resulting in a significant genotype x condition interaction [F(2,14) = 27.1, *p* < 0.0001]. However, as defined by a post-hoc Tukey–Kramer test, this reduction was not significant.

### Mice lacking a circadian clock show rhythmic daily locomotor activity but arrhythmic daily light environment sampling behaviour

**Fig. 5:**
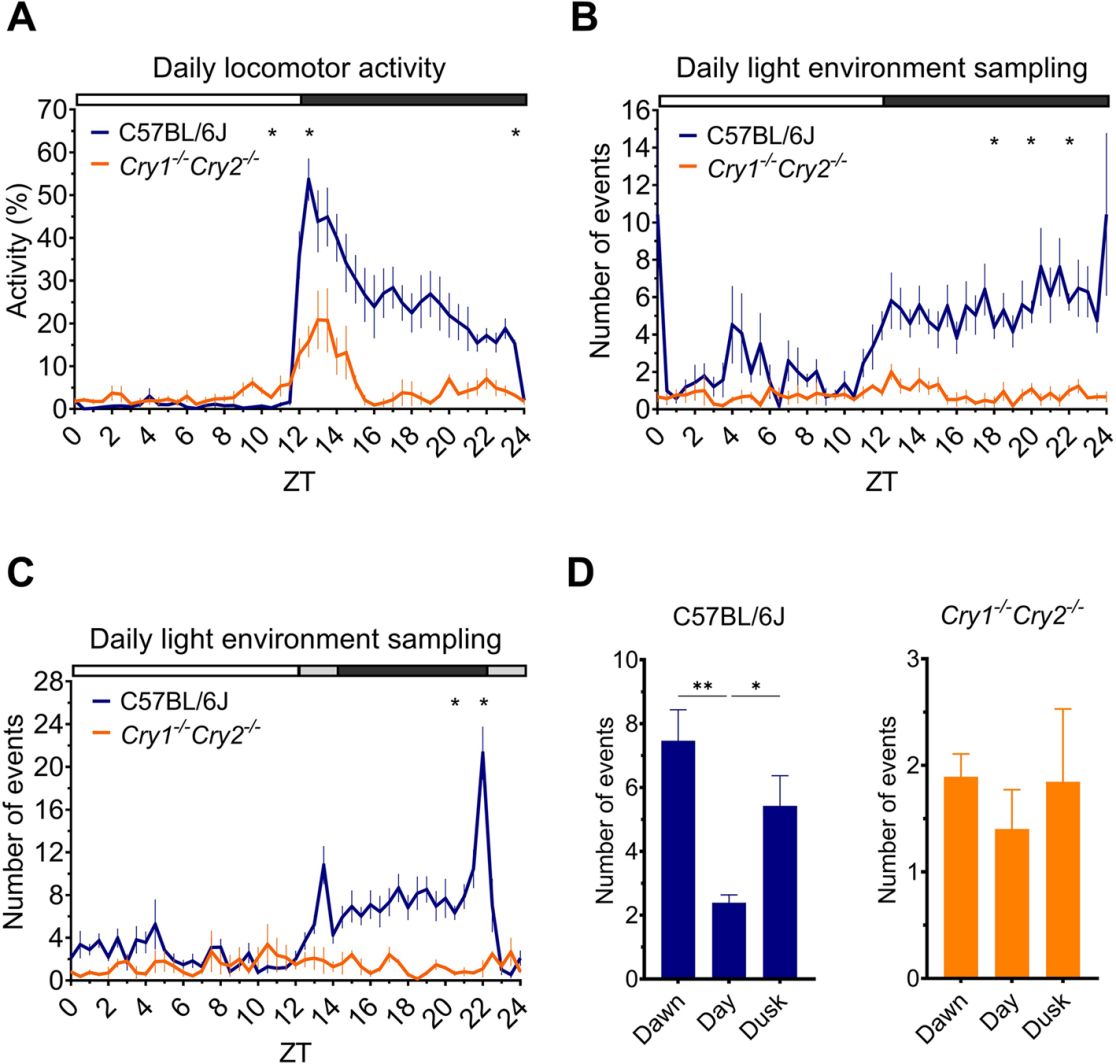
The circadian clock is important for the timing of light environment sampling behaviour. In all subplots C57BL/6J animals are shown in blue and *Cry1*^*−/−*^*Cry2*^*−/−*^ animals shown in orange. **A** Daily locomotor activity profile of C57BL/6J and *Cry1*^*−/−*^*Cry2*^*−/−*^ animals housed with a nestbox under a 12:12 h LD cycle, calculated using the main cage PIR. **B** Daily light environment sampling profile of C57BL/6J and *Cry1*^*−/−*^*Cry2*^*−/−*^ animals under a 12:12 h LD cycle. **C** Daily light environment sampling profile of C57BL/6J and *Cry1*^*−/−*^*Cry2*^*−/−*^ animals under a 12:2:8:2 h LD cycle. **A**-**C** * *p* < 0.05, between genotype comparison. White, grey and black bar shows timing of light, light ramp and dark, respectively. **D** Number of light environment sampling events occurring during dawn (ZT22-0), the day (ZT0-12) and dusk (ZT12-14) by C57BL/6J and *Cry1*^*−/−*^*Cry2*^*−/−*^ animals (note different scales). **** *p* < 0.001, ** *p* < 0.01, * *p* < 0.05, time of day comparisons. All results reported as mean across mice and days, ± SEM

Both C57BL/6J (Fig. 5A, blue) and *Cry1*^*−/−*^*,Cry2*^*−/−*^ mice (Fig. 5A, orange) show rhythmic patterns in locomotor activity across time [significant main effect of ZT, F(2.6,18.0) = 14.83, *p* < 0.0001, two-way repeated-measures ANOVA and post-hoc Bonferroni test]. This is to be expected, as although *Cry1*^*−/−*^*,Cry2*^*−/−*^ mice lack a circadian clock they are still able to show rhythmic locomotor activity patterns under an LD cycle [61] due to the direct activity-suppressing effects of light on activity in mice, known as negative masking [62]. Although locomotor activity rhythms in *Cry1*^*−/−*^*,Cry2*^*−/−*^ mice had a lower amplitude than in C57BL/6J control counterparts, they followed a similar pattern resulting in no significant main effect of genotype [F(1,7) = 10.03, *p* = 0.0158]; but a significant ZT x genotype interaction [F(48,336) = 6.4, *p* < 0.0001] since light phase activity was slightly higher in *Cry1*^*−/−*^*,Cry2*^*−/−*^ mice but lower than C57BL/6J mice during the dark phase.

However, despite daily locomotor activity being rhythmic in *Cry1*^*−/−*^*,Cry2*^*−/−*^ mice, light environment sampling behaviour is arrhythmic (Fig. 5B – orange), as confirmed by chi-squared periodogram analysis (data not shown). This is in contrast to the rhythmic pattern exhibited by the C57BL/6J mice (Fig. 5B – blue) (no significant main effect of ZT [F(2.8,19.8) = 1.8, *p* = 0.1760], but a significant main effect of genotype [F(1,7) = 14.6, *p* = 0.0065] and genotype x ZT interaction [F(48,336) = 1.73, *p* = 0.0030], two-way repeated-measures ANOVA and post-hoc Bonferroni test). Together, this suggests that the circadian clock is important for determining the timing of light environment sampling behaviour. Moreover, these data show that arrhythmicity in mice lacking a circadian clock can be detected under a LD cycle, by measuring light environment sampling instead of locomotor activity.

### Mice lacking a circadian clock do not show peaks in light environment sampling behaviour at twilight

The crepuscular peaks in light environment sampling behaviour seen in C57BL/6J mice under a ramped LD cycle are abolished in mice lacking a circadian clock (Fig. 5C). This difference in light environment sampling across time between genotypes is statistically significant (significant main effect of genotype [F(1,7) = 29.7, *p* = 0.0010] and ZT [F(4.4,30.9) = 4.8, *p* = 0.0032], as well as genotype x ZT interaction [F(48,336) = 5.5, *p* < 0.0001]; two-way repeated-measures ANOVA and post-hoc Bonferroni test). The mean number of light environment sampling events occurring during dawn (ZT22-0), the day (ZT0-12) and dusk (ZT12-14) were calculated (Fig. 5D). Night time (ZT14-22) was not included since there is no light at this time to sample. A significant main effect of ZT [F(1.3,12.6) = 9.2, *p* = 0.0071] and genotype [F(1,10) = 31.0, *p* = 0.0002] were observed (two-way repeated-measures ANOVA and post-hoc Bonferroni test). There was also a significant ZT x genotype interaction [F(2,20) = 12.2, *p* = 0.0003], demonstrating that light environment sampling varies across twilight and the day differently between C57BL/6J and *Cry1*^*−/−*^*,Cry2*^*−/−*^ mice. A post-hoc Bonferroni test highlighted significantly higher levels of light environment sampling events at dawn (*p* = 0.0069) and dusk (*p* = 0.0477) compared to during the day, in C57BL/6J mice (Fig. 5D, left panel), but no significant differences in *Cry1*^*−/−*^*,Cry2*^*−/−*^ mice (Fig. 5D, right panel). This adds further support to the idea that the circadian clock is important for regulating the timing of light environment sampling behaviour, and therefore the amount and type of light available to an organism for photoentrainment.

## Discussion

Here we take an ecologically guided approach to circadian entrainment by providing the opportunity for mice to self-modulate their light exposure. We demonstrate that under this paradigm C57BL/6J mice will significantly reduce their daily light exposure to less than an hour across the 24 h period. This reduction mirrors previous literature on nocturnal den-dwelling flying squirrels, where animals housed with simulated dens only exposed themselves to a few minutes of light each day [36, 37]. DeCoursey observed that some flying squirrels spent the entire light phase in the dark nest, and free-run for several days until their activity onset advanced into the light phase [36, 37]. In the current study, all animals showed robust entrainment which is consistent with prior studies in mice [53]. Refinetti [53] represents the only previous study to investigate light environment sampling behaviour in mice and suggests no significant effect of being able to self-modulate light exposure on photoentrainment. However, in this study locomotor rhythms were primarily measured using wheel running behaviour, under square wave LD cycles. Wheel running only provides a measure of voluntary activity and under some conditions is known to influence behaviour and entrainment [63–65]. Here we measure activity across the cage, atrium and nest at a high resolution (1 s) and show that it is only under more naturalistic ramped twilight conditions that crepuscular light sampling behaviour is observed.

Given the photophobic behaviour of mice [66, 67] a reduction in light exposure when a dark nestbox is available is not surprising. However, the extent of the reduction is striking and illustrates a preference for light avoidance that is rarely accounted for in circadian studies and rodent husbandry. Light has potent effects on physiology and behaviour more widely, including on metabolism, hormone regulation and pain responses [68]. Our results therefore raise the question of whether enforced light exposure under standard laboratory conditions may result in behavioural and physiological effects that do not occur in the wild, and are largely unaccounted for in experimental design and analyses. A recent expert working group recommended that laboratory animals should have the opportunity to escape light by retreating to a shelter [69]. Although shelter enrichment such as red plastic or cardboard houses are often used, these reduce rather than abolish light exposure and are unlikely to reduce light exposure below the threshold for entrainment [14, 27, 70, 71].

The motivation for animals to use the nestbox may in part be light avoidance. However, the regular use of the nestbox during the dark phase (Fig. 2D) suggests that light avoidance alone cannot explain this. A nestbox may aid in behavioural thermoregulation, since standard laboratory temperatures are ~ 8 °C below the thermoneutral zone of 30 °C in mice [72, 73]. However, preliminary thermal imaging of nests indicate comparable increases in temperatures when built inside (Additional file 5: Fig. S5B) and outside of a nestbox (Additional file 5: Fig. S5C), suggesting that a nestbox does not contribute substantially to thermoregulation. However, this could differ with the varying dimensions of mice [74, 75] relative to the nestbox volume. Extending our paradigm to incorporate a transparent nestbox control, as well as temperature modulation [76] and different nesting materials [77], could clarify how different motivations interact to regulate this behaviour [78]. Thigmotaxis, an innate preference demonstrated by rodents to seek shelter or move in contact with walls instead of exposing themselves to an aversive open area [79, 80] could also be another factor driving use of the nestbox.

In addition to simply using the nestbox, mice showed extensive light environment sampling behaviour (Fig. 2D) distinct from overall locomotor activity (Fig. 2C). Light environment sampling behaviour was defined as the movement from the nest to atrium section of the nestbox, since the external light environment could be detected from here (Fig. 1C), mirroring the movement to a burrow entrance. The further reduction in light exposure when forage mix was added (Fig. 2A) suggests that the need to feed may be a key motivator behind light environment sampling behaviour. In support of this hypothesis, Decoursey [37] showed that 69% of time spent outside of the nestbox during the light phase by flying squirrels involved feeding and drinking.

Regardless of the motivation, our data demonstrates that behaviour can directly determine the timing of light exposure, with implications for photoentrainment. Under a natural light environment this would directly influence the intensity and spectral composition of light exposure, which vary across the 24 h cycle [28, 81]. Under a ramped LD cycle, the pattern of light exposure is consistent across days (Additional file 3: Fig. S3), with clear peaks in light environment sampling behaviour at twilight in C57BL/6J mice (Figs. 3A and 5C). This provides experimental evidence to support the hypothesis that nocturnal burrow-dwelling rodents sample their light environment more at twilight, thereby regulating the timing of activity with respect to the phase response curve (PRC) [38], as under the discrete entrainment model [82]. However, evidence suggests that models of photoentrainment cannot always be generalised between species with different natural histories, and therefore different patterns of light exposure. For example, the European ground squirrel entrains without ever seeing twilight [83], and a subterranean rodent, the tuco-tuco, can entrain to a single 1 h light pulse delivered randomly within the day [84].

Since all other conditions remained constant between the square wave and ramped LD cycles, the dynamically changing light levels almost certainly provide a more salient stimulus for light environment sampling behaviour compared to abrupt square-wave changes in light exposure. This suggests that rods and cones may play an important role in regulating this behaviour since they have an increased temporal resolution compared with melanopsin [24]. S-cones may be particularly important under natural twilights, which are short-wavelength enriched. Indeed, recent data has demonstrated the light-seeking and activity promoting effects of selective S-cone activation [85] which would align with increased light environment sampling behaviour. In addition, levels of S-cone opic lux have been shown to correlate most strongly with activity onset under a ramped white fluorescent LD cycle compared to other photoreceptor α-opic lux [86]. The increased light environment sampling at dawn compared with dusk is intriguing (Figs. 3A and 5C). This could result from light levels increasing against a dark background at dawn—generating greater stimulus contrast against the dark-adapted retina compared to at dusk, where a light-adapted retina must detect decreasing light levels. Given these different photosensory tasks of detecting dawn and dusk the photoreceptors involved may even differ – with rods playing more of a role at dawn and cones more at dusk. Moreover, the presence of a nestbox may change the nature of this task, for example, by decreasing light adaptation at dusk. The detailed role of different photoreceptors in mediating light environment sampling behaviour remains to be fully explored [81, 87–89].

By understanding and recreating the natural light environments of model organisms we may be able to better account for the differences we see between laboratory and field studies in circadian neuroscience [45]. Flôres [80] tackled this issue by simulating the natural pattern of light exposure of the tuco-tuco in the laboratory. In our study this is taken further by giving animals the opportunity to self-modulate their pattern of light exposure; an approach which may yield greater mechanistic insights into photoentrainment under naturalistic conditions. However, comparisons between our data and house mouse activity patterns in the wild are limited by the small number of studies published on the latter, and methodological differences [30, 35, 90]. Whilst our locomotor activity data mirrors wild studies, with activity peaking in the 2 h following sunset and declining across the remainder of the night [30], these studies do not characterise light exposure or light environment sampling behaviour, which we know differ from overall locomotor activity (Fig. 1C, D). Conversely, our studies were performed under constant temperature conditions, food and water provided ad libitum, and with no risk of predation, which does not accurately recapitulate natural conditions [34].

**Fig. 6:**
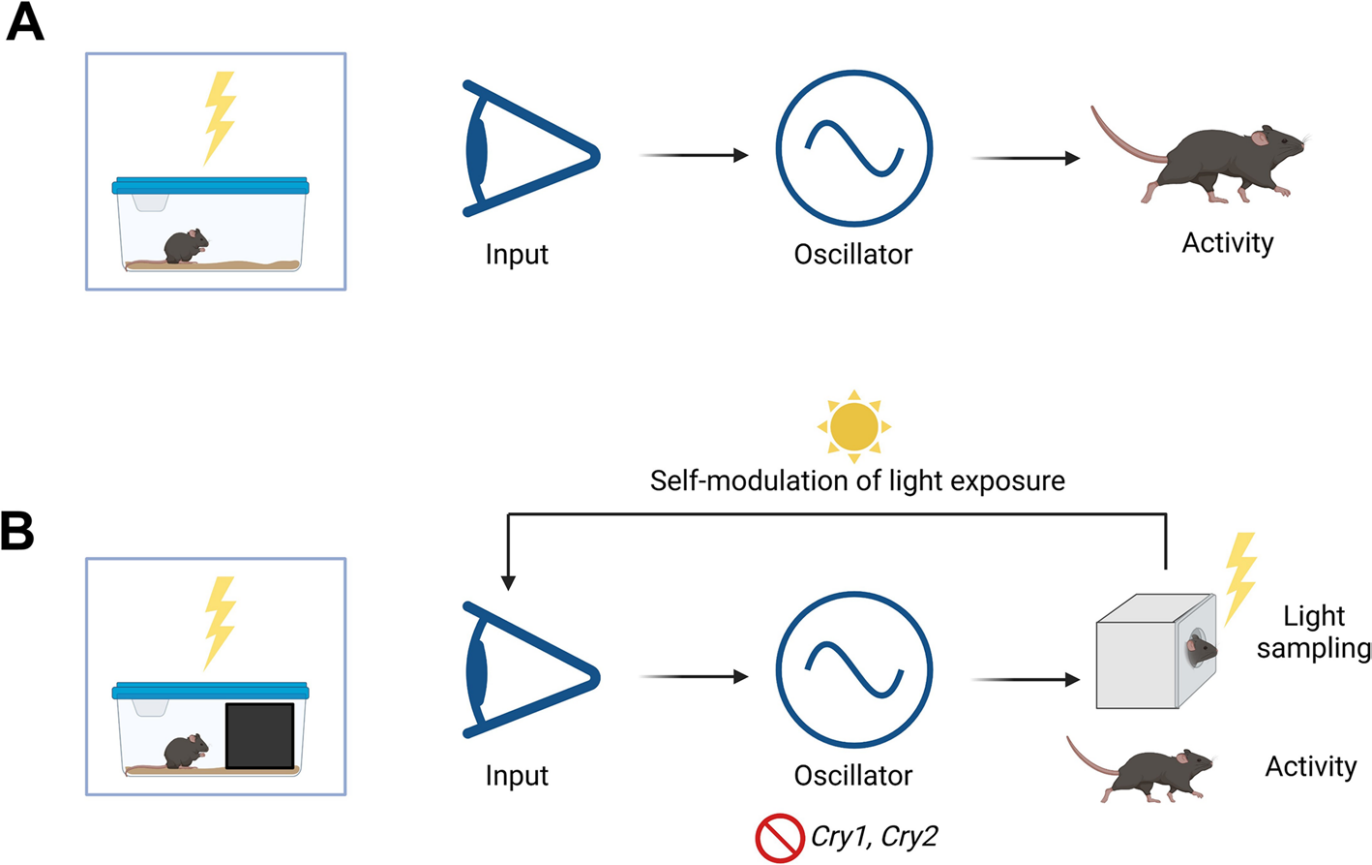
Photoentrainment schematic under standard laboratory conditions **(A)** and with a dark nestbox **(B)**, illustrating the feedback loop between behaviour, light and the circadian clock when a nestbox is present

Here we also investigated the mechanistic basis of light environment sampling behaviour. We demonstrate for the first time that light environment sampling behaviour is arrhythmic in mice lacking a circadian clock (*Cry1*^*−/−*^*Cry2*^*−/−*^) (Fig. 5B, C and D), even though locomotor activity remains rhythmic under a LD cycle due to masking (Fig. 5A) [62]. Along with the regular pattern of daily light environment sampling behaviour shown by wildtype mice (Additional file 3: Fig. S3), this suggests that the circadian clock plays an important role in providing a consistent pattern of light exposure for photoentrainment. This may help to optimise photoentrainment by preventing inappropriate phase shifts in activity that might arise if the timing of daily light exposure is highly variable [23]. In this way, light environment sampling behaviour is a downstream output of the SCN that provides an important feedback to modulate light input to the SCN clock (Fig. 6B). These findings illustrate how behaviour can influence seemingly independent processes. This feedback loop between behaviour, light and the circadian clock (Fig. 6B) cannot occur under standard laboratory conditions (Fig. 6A), showing how the provision of a dark nestbox can enable the more complex features of photoentrainment to be studied.

The model proposed in Fig. 6 will undoubtedly be more complex, with several potential explanations as to why CRY-deficient mice show arrhythmic light environment sampling behaviour under an LD cycle. These may be both behavioural and physiological, and could include differences in locomotor activity and exploratory behaviour [56], sleep/wake changes [91], metabolic changes [92–94], time-place learning [95], retinal gating to the SCN [57], and photic sensitivity [27, 96, 97]. These additional phenotypes influencing circadian physiology and behaviour could also be candidate zeitnehmers (meaning ‘time taker’) [98] since they are under circadian control but feedback to impact the entrainment process by influencing light sampling behaviour [99]. Of these factors, direct alterations to photic sensitivity in CRY-deficient mice may be of particular relevance to light environment sampling behaviour. Retinal development can be altered in mice lacking core clock genes, as demonstrated by the lack of an S-cone opsin retinal gradient in mice lacking *Bmal1* [100], and since CRY1 is expressed in both mouse cone photoreceptors it raises the question as to whether the abolition of cryptochrome may alter cone development and sensitivity [96, 97]. It is therefore possible that arrhythmic light sampling behaviour may occur in CRY-deficient mice due to direct alterations to photic sensitivity associated with this model, in addition to behavioural and physiological circadian defects.

Beyond enhancing our understanding of photoentrainment, the nestbox paradigm presented here may also have other potential applications. The decision making behaviour we describe in the “go” vs “no go” responses (Fig. 3C, D) is likely to reflect an internal conflict between the need to explore and access food and water versus staying in the safety of the dark nestbox [78]. A long-term home-cage test with ethological relevance [101] might provide a measure of anxiety and avoid issues such as handling that can alter behavioural measures [102]. In addition, Wiedenmayer [103] showed that a dark chamber with a tunnel entrance is sufficient to prevent stereotypic digging behaviour in gerbils, whilst simply providing digging substrate was not. These findings suggest that providing a dark nestbox may serve to reduce stereotypic locomotor behaviour in rodent models [49]. Further studies are required to explore the wider benefits of home cage nest boxes for laboratory mouse behaviour.

Finally, consideration of light exposure patterns is also of relevance to human circadian studies, as the lighting conditions experienced by humans in modern societies are very different to natural light exposure, with low light intensity during the day and artificial light at night extending our light phase beyond sunset [104–106]. Indeed, bright light exposure during the daytime can decrease the sensitivity of the circadian system to dim light in the evening [107]. Understanding how behaviour modifies the timing, intensity and wavelength of light exposure may provide opportunities for modifying human behaviour and environments to promote the establishment of healthier lighting exposure patterns [105].

## Conclusions

Here we provide a simple experimental paradigm that enables mice to self-modulate their light exposure, thereby addressing a key difference between the natural and laboratory light environment of mice [30]. In parallel, this approach allows quantification of locomotor activity, circadian entrainment, light environment sampling behaviour and decision making within a long-term home-cage environment. We show that when given the opportunity, mice will significantly reduce their light exposure and exhibit light environment sampling behaviour, with peaks at twilight under a ramped LD cycle. This highlights the important role of behaviour in modifying the signals available for photoentrainment under more naturalistic environments, that is often overlooked in laboratory studies. Moreover, we show that light environment sampling behaviour is arrhythmic in mice lacking a circadian clock, demonstrating an important role for the circadian system in regulating the timing, intensity and potentially the wavelength of light exposure. Collectively, our data illustrate how under natural conditions light environment sampling behaviour forms an important feedback on circadian entrainment, rather than being a simple output of the circadian clock.

## Methods

### Animals and housing conditions

For the first light environment sampling study, 12 C57BL/6J mice were used (Envigo, Blackthorn, United Kingdom, RRID:IMSR_ JAX:000664). They were tested in two cohorts of 6 animals, run at separate times. To examine the role of the circadian clock in regulating light environment sampling behaviour, 6 *Cry1*^*−/−*^*,Cry2*^*−/−*^ mice (lacking a circadian clock [61]) and 6 congenic C57BL/6J controls were used (Envigo, Blackthorn, United Kingdom, RRID:IMSR_ JAX:000664). Founder *Cry1*^*−/−*^*,Cry2*^*−/−*^ mice were a kind gift from Gijsbertus T. J. van der Horst (Erasmus MC, Rotterdam, Netherlands) [61]. All animals were aged ~ 10 weeks at the start of the control week and all groups were sex balanced. All animals were singly housed with ad libitum access to food and water, located in a food hopper and water bottle in the main cage (i.e. outside the nestbox) (Fig. 1C). The substrate of the cages were sawdust shavings (eco-pure aspen chips 4, Datesand; UK) and we observed no evidence of animals blocking the entrance of the nestbox*.* Sizzle nest was provided as a nesting material throughout the experiment (Sizzlenest, Datesand; UK). It was placed in the main cage at the beginning of the experiment, and the majority of mice moved the sizzlenest into the nestbox.

Cages were placed within light-tight ventilated chambers (LTCs) equipped with multiple WiFi controlled cool-white (4500 CCT) light-emitting diodes (LEDs) (LIFX light-strip; LIFX, Cremorne, Australia), providing a light level of 200 photopic lux (5 S-cone opic lux, 170 melanopic lux, 169 rhodopic lux and 170 M-cone opic lux) throughout the light phase; calculated using the Rodent Toolbox. The spectral power distribution of the cool-white LED consisted of a high, narrow peak at ~ 450 nm and a lower, broader peak at ~ 560 nm (Additional file 7: Fig. S7; spectral power distribution (SPD) reported in supplementary materials), measured using a calibrated Ocean Optics USB2000 + Spectrophotometer (Ocean Insight, Oxford, United Kingdom). The temperature of the animal holding room was maintained at 19–21 °C. All experimental procedures were conducted at the University of Oxford, England, in accordance with the United Kingdom Animals (Scientific Procedures) Act 1986 under Project License PP0911346 and Personal License I82616702. All procedures were in accordance with the University of Oxford Policy on the Use of Animals in Scientific Research.

### Experimental design

All animals, in all experiments, were exposed to the same initial sequence of conditions (Table 1). Prior to the onset of the experiment, all mice were habituated to a reverse square wave 12 h:12 h LD cycle (lights on at 19:00 and lights off at 07:00) for 2 weeks. This LD cycle remained constant for weeks 1–6 of the experiment. Following habituation there was a control week of standard laboratory conditions, before a nestbox was added to each cage for 2 weeks (Fig. 1A). The first week was to allow habituation to the nestbox and only the second week of recordings were used in analysis. The nestbox remained in place for the remainder of the experiment. Throughout week 4, forage mix (LBS forage mix; LBS Biotechnology, UK) was added to the cage floor during daily welfare checks, in addition to standard food in the food hopper (Fig. 1C), to examine the effect of being able to take food back to the nestbox on activity and entrainment. In weeks 5 and 6, a 12:2:8:2 h cycle was used to explore the effect of a more naturalistic LD cycle. This was composed of a 12 h light phase (170 melanopic lux of cool-white LED) followed by a 2 h ramp of decreasing light intensity, an 8 h dark phase and a 2 h ramp of increasing light intensity. Both ramps followed an exponential pattern, with light intensity measuring 16 photopic lux at 1 h into the ramp. The timings of the 12:2:8:2 h ramped LD cycle were based on the length of daylight and twilight naturally occurring at the spring and vernal equinoxes in Oxford, UK [108]. The equinox LD cycle was used since it is an intermediate LD cycle, with the length of the light phase being in-between those of the summer and winter solstices. The first week was to allow habituation to the ramped LD cycle and only the second week of recordings were used in analysis. All animals in the second experiment (*Cry1*^*−/−*^*,Cry2*^*−/−*^ vs. C57BL/6J controls) were exposed to an additional 2 weeks of constant dark (DD) at the end, to check for expected free-running locomotor activity patterns in C57BL/6J controls and arrhythmicity in *Cry1*^*−/−*^*,Cry2*^*−/−*^. The first week of DD was for habituation, and only the second week was used for analysis (data not shown).

**Table 1:** Experimental design timeline. All animals in all studies were exposed to week 1–6 conditions. The 12 animals in the second study (Cry1^−/−^,Cry2^−/−^ vs. C57BL/6J) were exposed to an additional two weeks (weeks 7 and 8, in bold).

WEEK	LIGHTING	CONDITION	PURPOSE
1	Reverse 12:12 h LD cycle	Standard	Control
2	Reverse 12:12 h LD cycle	Nestbox	Nestbox habituation
3	Reverse 12:12 h LD cycle	Nestbox	Nestbox data collection
4	Reverse 12:12 h LD cycle	Nestbox + forage mix	Nestbox + forage mix
5	Reverse 12:2:8:2 h LD cycle	Nestbox	sRamped LD habituation
6	Reverse 12:2:8:2 h LD cycle	Nestbox	Ramped LD data collection
**7**	**Constant dark (DD)**	**Nestbox**	**DD habituation**
**8**	**Constant dark (DD)**	**Nestbox**	**DD data collection**

### Locomotor activity monitoring

From week 2 onwards, all cages were fitted with a nestbox. The nestbox was designed in Blender (v3.6.4 LTS, https://www.blender.org/), sliced in Ultimaker Cura (v5.4.0) and 3D printed (Ultimaker S3 printer; designs available in Additional file 8) in black ABS plastic (Ultimaker 1621). The nestboxes (Fig. 1B) were comprised of two sections – a base and a top which were glued together, and all walls were 5 mm thick. The base (90 mm(H) x 100 mm(W) x 140 mm(D)) had two internal sections, a larger nesting section at the back (internal dimensions of 85 mm(H) x 90 mm(W) x 80 mm(D)) and a smaller atrium section at the front (internal dimensions of 85 mm(H) x 90 mm(W) x 45 mm(D)), a design influenced by Kallmyer [109]. Between the nesting section and the atrium, and the atrium and the main cage, there were staggered open archways measuring 30 mm(H) x 40 mm(W). This allowed free movement between compartments, and for light to be detected from the atrium entrance whilst ensuring a dark nesting section. As measured by an XL-500 BLE Spectroradiometer (dynamic range = 0.1 to 40,000 photopic lux; NanoLambda, Korea) the light level in the nesting section was < 0.1 photopic lux and the atrium section ranged from < 0.1 to 0.4 photopic lux, with distance from the entrance into the main cage (Fig. 1C). The top section of the nestbox was a 5 mm thick wall (21 mm(H) x 100 mm(W) x 140 mm(D)) and was glued on top of the nestbox base to ensure the structure was flush with the top of the cage.

A passive infrared sensor (PIR) [110] was fitted into a 10 mm diameter hole in the centre of each of the nesting and atrium compartments, and a third PIR sensor was fitted 22 cm above each cage. Each PIR sensor records movement as a binary measurement every 10 ms and combines this data across 1 s bins, outputting a percentage activation of the sensor across every 1 s epoch. This enabled quantification of locomotor activity circadian entrainment, decision making and light environment sampling behaviour. Locomotor activity was defined as movement in the main cage, outside of the nestbox. Light environment sampling behaviour was defined as a movement within the nestbox—specifically from the dark nest to the atrium compartment, from which the animal is able to detect the external light environment, as at the burrow entrance. We refer to it as light *environment* sampling, where the light environment could be light or dark, rather than simply light sampling since during the dark phase there is no light to sample. Every 6 PIRs were connected to a 6-port Arduino (Arduino Uno R3). 3 Arduinos were subsequently connected to a Raspberry Pi (Raspberry Pi 3 B) and Node Red (v3.1.0) was used to collect and backup data on the Raspberry Pi. PIRs collected activity data every 1 s, as the standard 10 s data collection [110] did not provide a high enough temporal resolution to accurately track the animal as it moved between sensors. The light schedule of the LTCs was confirmed using a light-dependent resistor (LDR) connected to the PIR system.

### Data processing

Raw PIR activity and LDR data was processed in MATLAB (v.R2022b), ImageJ (v.1.53a, using the Actogram J plugin [111]) and Excel (v.2310). No significant differences between cohorts 1 and 2 of the first C57BL/6J light environment sampling study were found, so cohorts were processed and analysed together.

### Locomotor activity profiles

Raw main cage PIR activity and LDR data starting at ZT0 for 7 consecutive days (ZT = zeitgeber time; ZT0 = lights on, ZT12 = lights off) was smoothed with a 30 min moving average to generate daily activity profiles for each experimental condition (MATLAB).

### Location and light exposure

A MATLAB function *(location_finder.m)* was written to calculate the location (cage, atrium or nest) of each mouse at each 1 s time point. This function filled in the location of the mouse using the 3 PIR channels of activity data (cage, atrium, nest) to account for the mouse being present but immobile in a location. If all PIR channels were reading 0, then it moved back rows until it hit a value of > 0 in one of the columns. A value of 1 was assigned to this channel. This produced a dataset for each mouse, where 1 equalled present and 0 equalled not present, across every second at all three locations (cage, atrium, nest). Using the location data, daily light exposure could subsequently be calculated. This was defined as the time spent (hrs) in the main cage during the light phase. For the control week this is automatically 12 h, as there was no nestbox available.

### Light environment sampling behaviour and decision making

Analysis of light environment sampling behaviour and decision making was also based on the location data. A MATLAB function *(simplify_columns.m)* was written which took the location data and generated a new matrix, to ensure that only one sensor was active at a time (if a mouse moved across the three PIR sensors within 1–3 s, then two or three sensors would show activity at each time point, due to sensor lag). If all three location columns equalled 0, three 0 s were assigned to the new matrix. If all columns were 1, a 1 was assigned to the atrium column and a 0 to the nest and cage (since the mouse is moving from the cage to the nest, through the atrium, or vice versa). If two columns equalled 1, then it moved up rows until one of the rows equalled 0. A 0 was assigned to this column and a 1 to the other column. A MATLAB function *(transitions.m)* was written in MATLAB to then take the simplified data and create a new matrix identifying light environment sampling behaviour (defined as a nest to atrium transition, and assigned as ‘1’ in the new matrix), followed by either entry to the cage (a “go” decision, assigned as ‘2’) or a return to the nest (a “no-go” decision, assigned as ‘3’) (Fig. 3C). The sum of the “go” and “no go” transitions equalled the total number of light environment sampling events.

### Circadian parameters

Key circadian entrainment metrics were calculated as in [55], using activity data from the main cage PIR sensor. MATLAB was used to calculate light phase activity, dark phase activity, relative amplitude, inter-daily stability and intra-daily variability. The chi-squared periodogram power (Qp) [112] and activity onsets were calculated using inbuilt functions in Actogram J [111, 112].

### Statistical analysis

Statistical analysis and data visualisation were performed in MATLAB and Prism Graph-pad (v.9.5.0). All data is reported as mean across days and animals, ± SEM, and α = 0.05 was adopted in all analyses. All locomotor, light environment sampling and decision making daily profiles are visualised in 30 min bins. Any animals that did not routinely use the nestbox were removed from the analysis (2 C57BL/6J animals in the initial light environment sampling study, and 3 of the *Cry1*^*−/−*^*,Cry2*^*−/−*^ animals in the second experiment). The Greenhouse–Geisser correction was performed with all ANOVAs, unless otherwise stated. A post-hoc Tukey test was used when all pairwise comparisons were desired, and a Tukey–Kramer test where sample sizes were unequal; whilst a post-hoc Bonferroni test was used for a specific comparison between the control and experimental treatments. Further details on statistical tests used for each dataset are reported in the results section.

## Supplementary Information

Additional file 1: Fig. S1. The majority of C57BL/6J mice routinely use the nestbox. Daily light exposure (hrs) (mean across days ± SEM) of individual C57BL/6J mice across experimental conditions (control, nestbox, and nestbox + forage mix (‘ + forage mix’)), in the C57BL/6J light sampling study.

Additional file 2: Fig. S2. Other circadian entrainment parameters for C57BL/6J mice from the first light sampling study, across experimental conditions (control, nestbox, nestbox + forage mix (‘ + forage mix’). Reported as mean ± SEM. (A) Dark phase activity (%). (B) Intradaily variability. (C) Activity onset. (D) Activity onset variance (calculated as SD of activity onsets across days, across mice). (E) Circadian period. * *p* < 0.05, between condition comparisons.

Additional file 3: Fig. S3. Light environment sampling behaviour shows consistent patterns across days. Double plotted actograms of light environment sampling events across a week of a representative C57BL/6J animal under experimental conditions. (A) Nestbox available, 12:12 h LD cycle. (B) Nestbox and forage mix available, 12:12 h LD cycle. (C) Nestbox available, 12:2:8:2 h LD cycle. Actograms produced using MATLAB code, adapted from https://github.com/abubnys/GA_actograms.git.

Additional file 4: Fig. S4. Males and females show differences in locomotor activity but not light environment sampling behaviour, under square and ramped LD cycles. (A) Daily locomotor activity profile of females (green) and males (blue) under 12:12 h LD cycle. (B) Daily light environment sampling profile of females (green) and males (blue) under 12:12 h LD cycle. (C) Daily locomotor activity profile of females (green) and males (blue) under 12:2:8:2 h LD cycle. (D) Daily light environment sampling profile of females (green) and males (blue) under 12:2:8:2 h LD cycle. All results reported as mean across days and animals, ± SEM. * *p* < 0.05, between sex comparisons. White, grey and black bar shows timing of light, light ramp and dark, respectively.

Additional file 5: Fig. S5. Thermal images of nests and nestboxes (FLIR one pro, Teledyne FLIR). (A) No nest or animal present. Imaged immediately after removal of uninhabited nestbox. Nestbox always positioned in top left corner of cage (A,B). (B) Nest built within nestbox. Imaged immediately after removal of nestbox and vacation of nest by animal. (C) Nest built with no nestbox present. Imaged immediately after vacation of nest by animal. In all photos, the blue dot refers to the lowest temperature spot and the red dot to the highest temperature spot. Spots 1 and 2 show the temperature of the zone of interest and a comparison to the main cage away from the nest and nestbox.

Additional file 6: Fig. S6. Fewer *Cry1*^*−/−*^*Cry2*^*−/−*^ mice use the nestbox than C57BL/6J mice. Daily light exposure (hrs) (mean ± SEM) under experimental conditions (control, nestbox, nestbox + forage mix (‘ + forage mix’). (A) C57BL/6J. (B) *Cry1*^*−/−*^*Cry2*^*−/−*^*.*

Additional file 7: Fig. S7. Spectral power distribution (SPD) of cool white LED (4500 CCT) used throughout the experiment (LIFX light-strip; LIFX, Cremorne, Australia). 200 photopic lux, 5 S-cone opic lux, 170 melanopic lux, 169 rhodopic lux, 170 M-cone opic lux, measured using a calibrated Ocean Optics USB2000 + Spectrophotometer (Ocean Insight, Oxford, United Kingdom).

## Data Availability

The 3D printing files, raw datasets and analysis scripts are available at the following links: • Additional file 8: https://doi.org/10.6084/m9.figshare.26496571.v1 (3D printing files for nestbox). • Additional file 9: https://doi.org/10.6084/m9.figshare.26496538.v1 (raw data: C57BL/6J study). • Additional file 10: https://doi.org/10.6084/m9.figshare.26496073.v1 (raw data: CrydKO vs C57BL/6J). • Additional file 11: https://doi.org/10.6084/m9.figshare.26496052.v1 (MATLAB scripts: C57BL/6J). • Additional file 12: https://doi.org/10.6084/m9.figshare.26496064.v1 (MATLAB scripts: CrydKO vs C57BL/6J).

## Abbreviations

DD: Constant dark
h(s): Hour(s)
LD cycle: Light:dark cycle
PRC: Phase response curve
SCN: Suprachiasmatic nuclei
ZT: Zeitgeber time
λmax: Peak wavelength sensitivity
