# Supplementary figures and images for: Light sampling behaviour regulates circadian entrainment in mice
Source: BMC Biol. 2024 Sep 16;22:208. doi: 10.1186/s12915-024-01995-x (PMC11404008; doi:10.1186/s12915-024-01995-x)

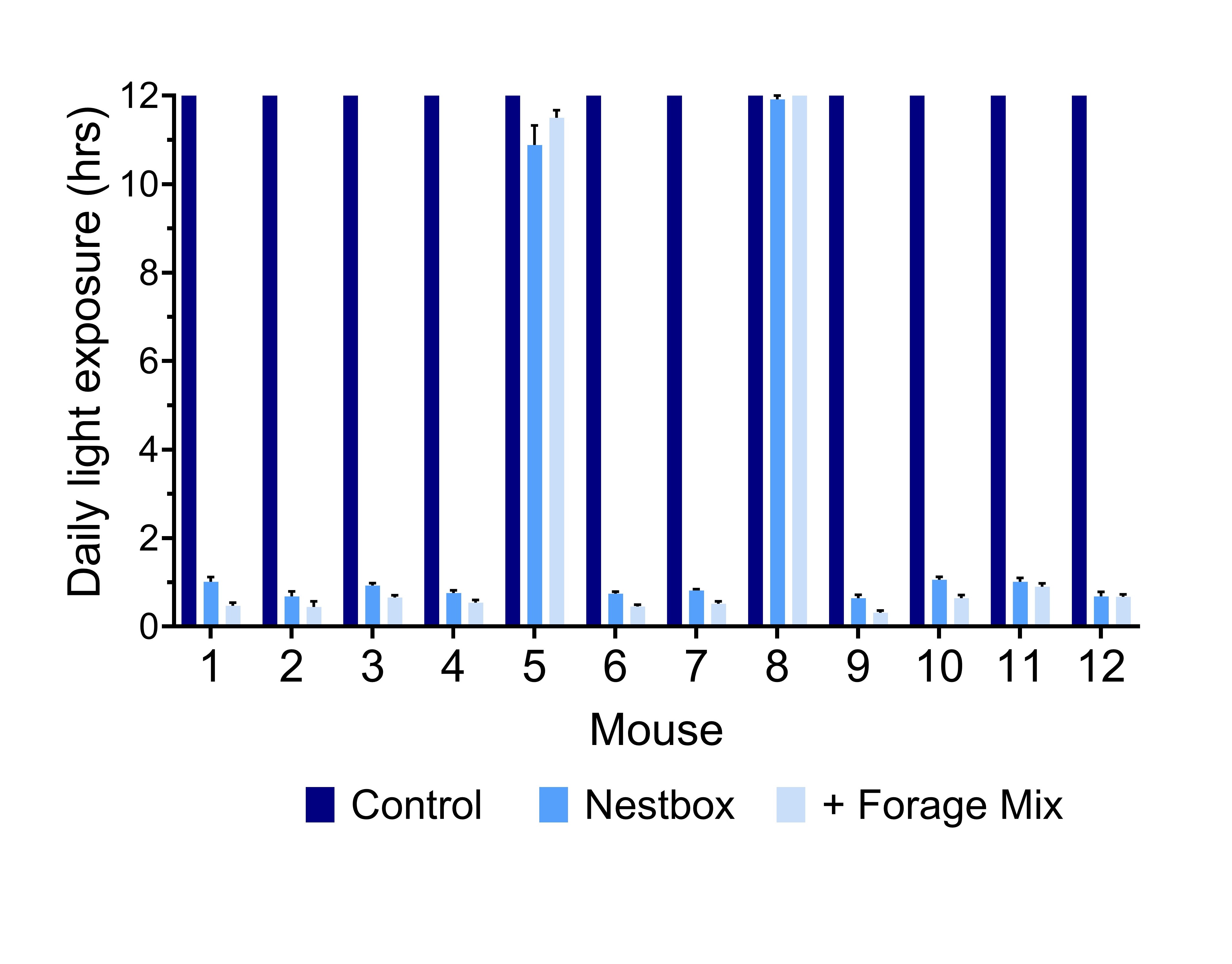

Supplement: Supplementary file 1 — Additional file 1: Fig. S1. The majority of C57BL/6J mice routinely use the nestbox. Daily light exposure (hrs) (mean across days ± SEM) of individual C57BL/6J mice across experimental conditions (control, nestbox, and nestbox + forage mix (‘ + forage mix’)), in the C57BL/6J light sampling study. [file 12915_2024_1995_MOESM1_ESM.jpg]

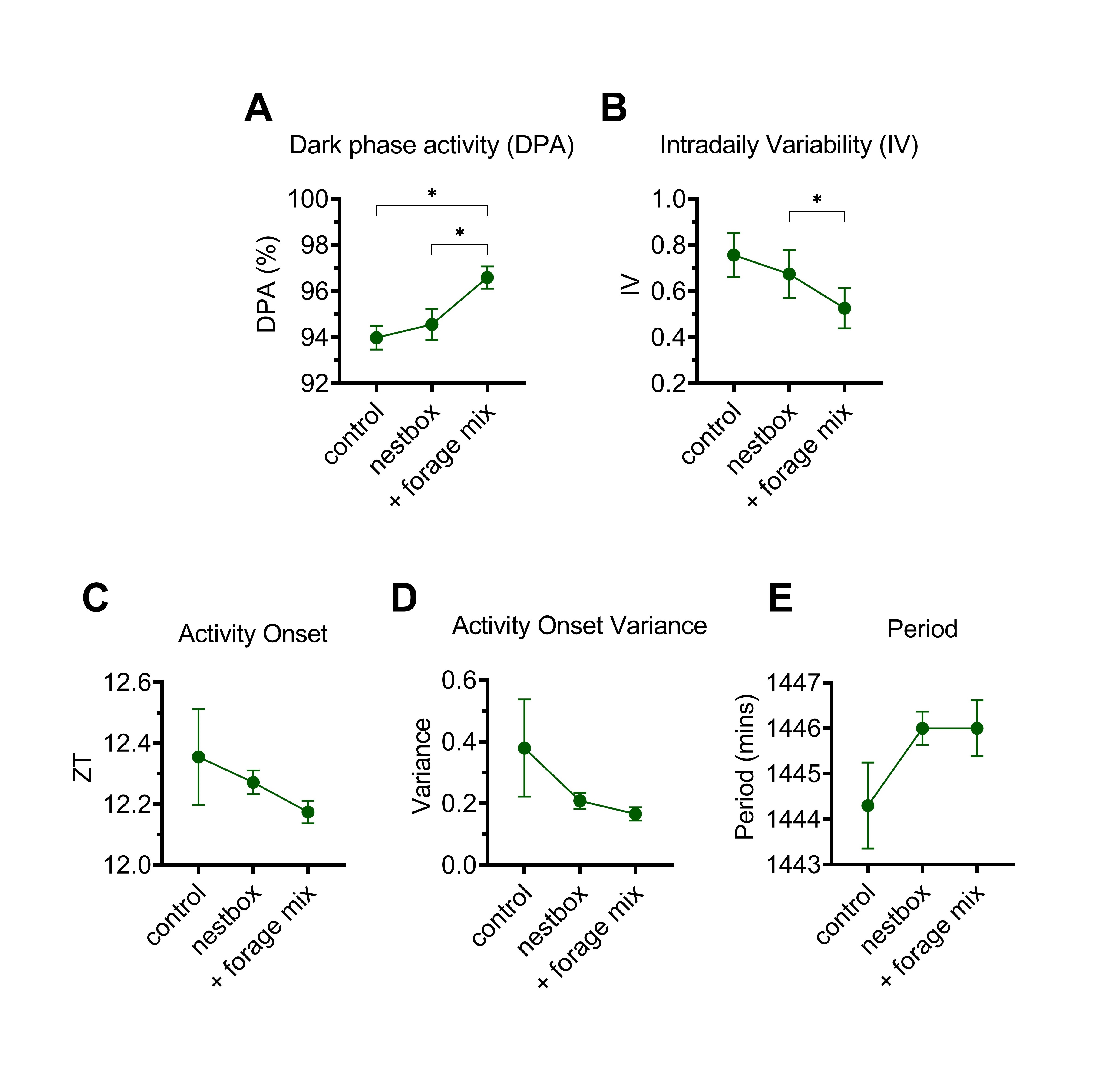

Supplement: Supplementary file 2 — Additional file 2: Fig. S2. Other circadian entrainment parameters for C57BL/6J mice from the first light sampling study, across experimental conditions (control, nestbox, nestbox + forage mix (‘ + forage mix’). Reported as mean ± SEM. (A) Dark phase activity (%). (B) Intradaily variability. (C) Activity onset. (D) Activity onset variance (calculated as SD of activity onsets across days, across mice). (E) Circadian period. * p < 0.05, between condition comparisons. [file 12915_2024_1995_MOESM2_ESM.jpg]

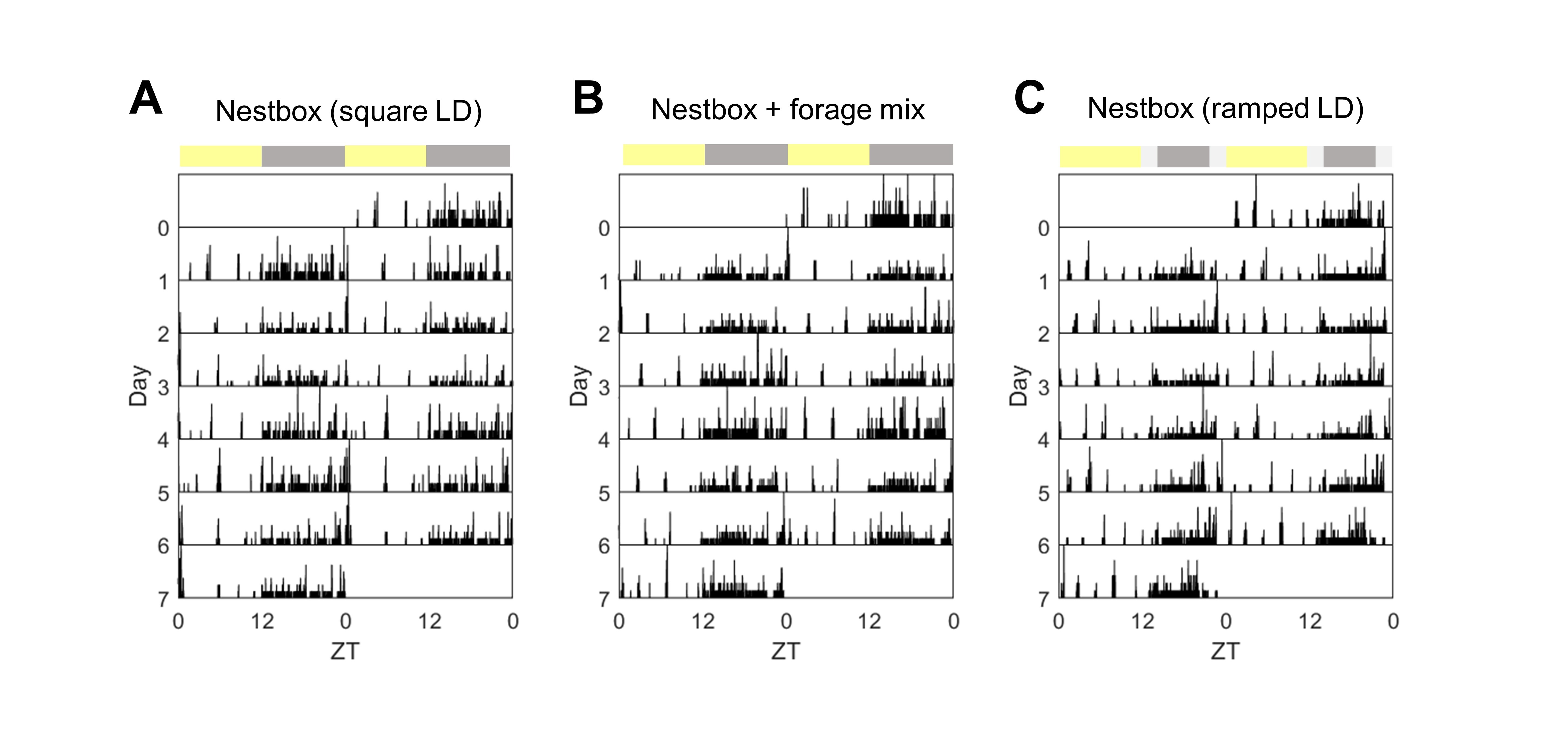

Supplement: Supplementary file 3 — Additional file 3: Fig. S3. Light environment sampling behaviour shows consistent patterns across days. Double plotted actograms of light environment sampling events across a week of a representative C57BL/6J animal under experimental conditions. (A) Nestbox available, 12:12 h LD cycle. (B) Nestbox and forage mix available, 12:12 h LD cycle. (C) Nestbox available, 12:2:8:2 h LD cycle. Actograms produced using MATLAB code, adapted from https://github.com/abubnys/GA_actograms.git. [file 12915_2024_1995_MOESM3_ESM.jpg]

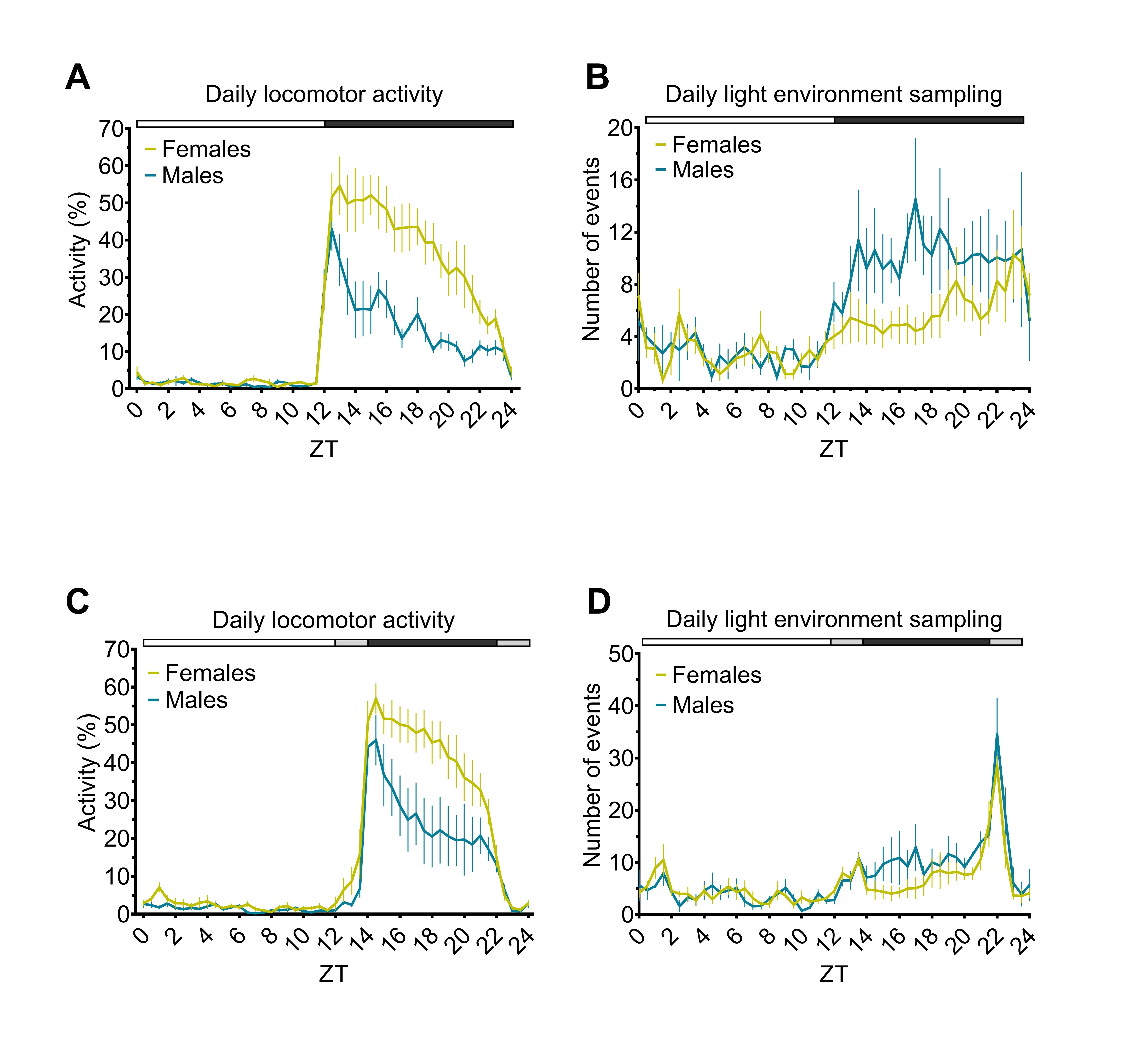

Supplement: Supplementary file 4 — Additional file 4: Fig. S4. Males and females show differences in locomotor activity but not light environment sampling behaviour, under square and ramped LD cycles. (A) Daily locomotor activity profile of females (green) and males (blue) under 12:12 h LD cycle. (B) Daily light environment sampling profile of females (green) and males (blue) under 12:12 h LD cycle. (C) Daily locomotor activity profile of females (green) and males (blue) under 12:2:8:2 h LD cycle. (D) Daily light environment sampling profile of females (green) and males (blue) under 12:2:8:2 h LD cycle. All results reported as mean across days and animals, ± SEM. * p < 0.05, between sex comparisons. White, grey and black bar shows timing of light, light ramp and dark, respectively. [file 12915_2024_1995_MOESM4_ESM.jpg]

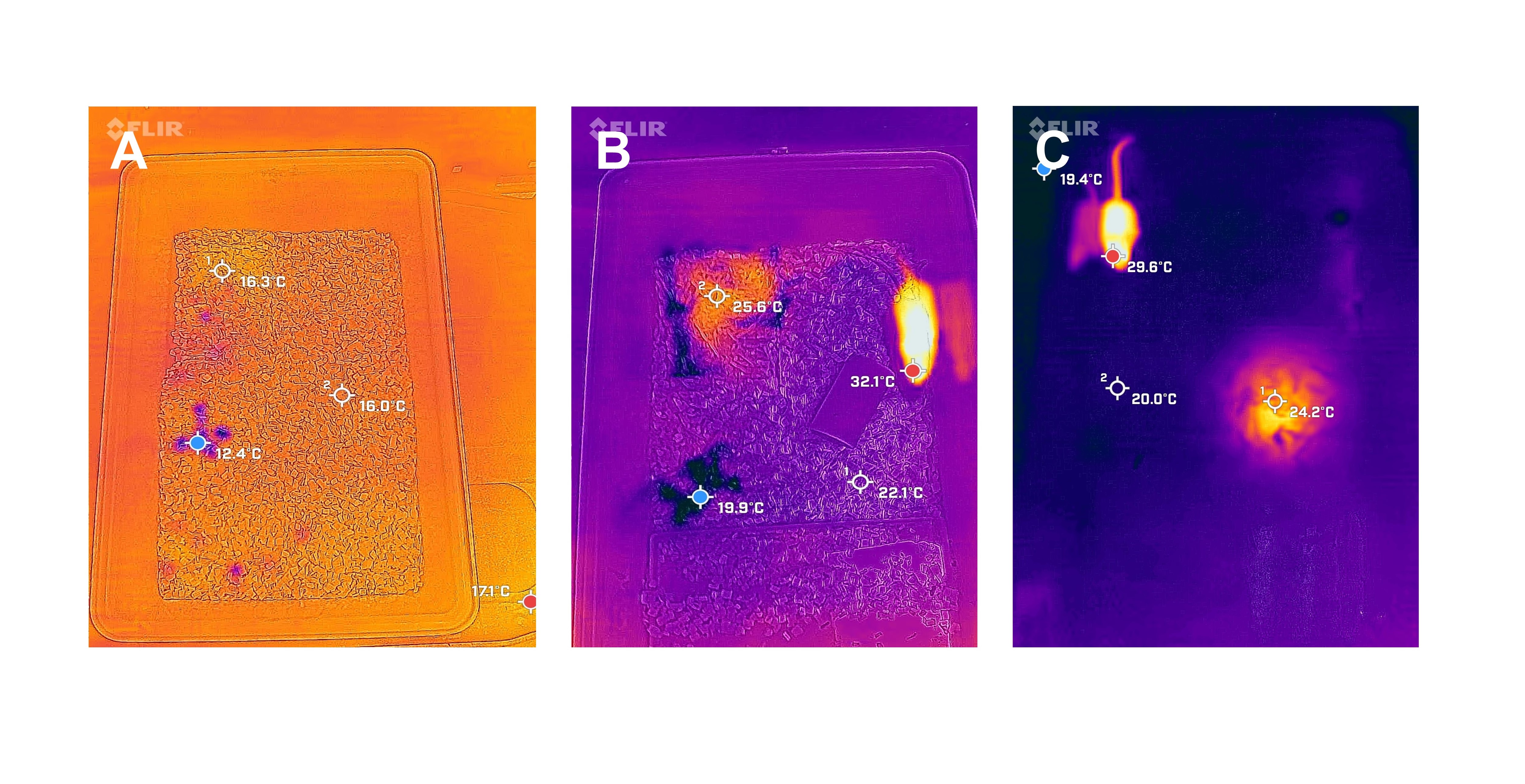

Supplement: Supplementary file 5 — Additional file 5: Fig. S5. Thermal images of nests and nestboxes (FLIR one pro, Teledyne FLIR). (A) No nest or animal present. Imaged immediately after removal of uninhabited nestbox. Nestbox always positioned in top left corner of cage (A,B). (B) Nest built within nestbox. Imaged immediately after removal of nestbox and vacation of nest by animal. (C) Nest built with no nestbox present. Imaged immediately after vacation of nest by animal. In all photos, the blue dot refers to the lowest temperature spot and the red dot to the highest temperature spot. Spots 1 and 2 show the temperature of the zone of interest and a comparison to the main cage away from the nest and nestbox. [file 12915_2024_1995_MOESM5_ESM.jpg]

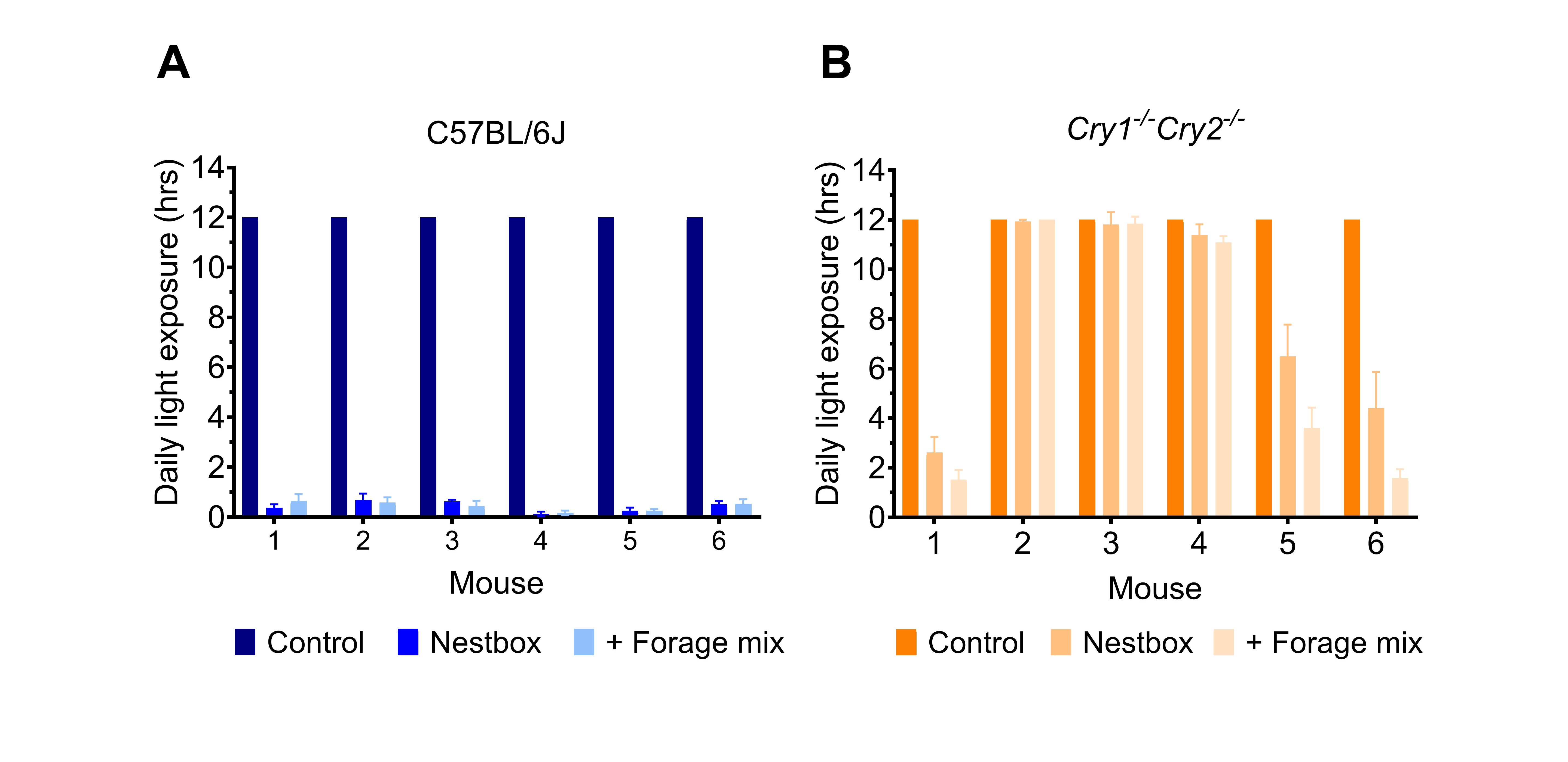

Supplement: Supplementary file 6 — Additional file 6: Fig. S6. Fewer Cry1−/−Cry2−/− mice use the nestbox than C57BL/6J mice. Daily light exposure (hrs) (mean ± SEM) under experimental conditions (control, nestbox, nestbox + forage mix (‘ + forage mix’). (A) C57BL/6J. (B) Cry1−/−Cry2−/−. [file 12915_2024_1995_MOESM6_ESM.jpg]

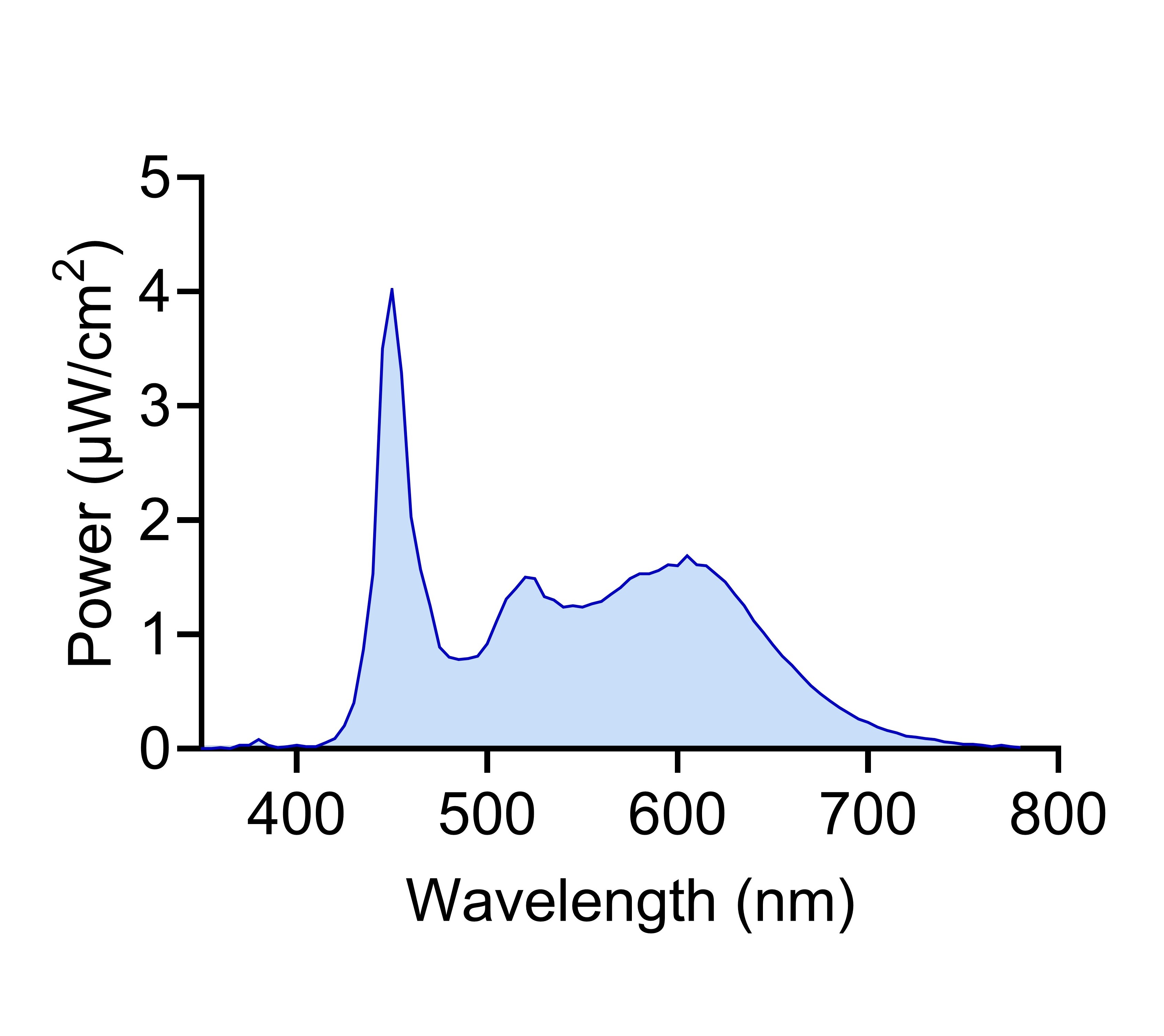

Supplement: Supplementary file 7 — Additional file 7: Fig. S7. Spectral power distribution (SPD) of cool white LED (4500 CCT) used throughout the experiment (LIFX light-strip; LIFX, Cremorne, Australia). 200 photopic lux, 5 S-cone opic lux, 170 melanopic lux, 169 rhodopic lux, 170 M-cone opic lux, measured using a calibrated Ocean Optics USB2000 + Spectrophotometer (Ocean Insight, Oxford, United Kingdom). [file 12915_2024_1995_MOESM7_ESM.jpg]
